# Long non-coding RNA AOC4P suppresses epithelial ovarian cancer metastasis by regulating epithelial-mesenchymal transition

**DOI:** 10.1186/s13048-020-00644-5

**Published:** 2020-04-25

**Authors:** Xiaojing Lin, Xiaoyan Tang, Tingting Zheng, Junjun Qiu, Keqin Hua

**Affiliations:** 1 Department of Gynecology, Obstetrics and Gynecology Hospital of Fudan University, 419 Fangxie Road, Shanghai, 200011 P.R. China; 2 Reproductive Medicine Center, The First Affiliated Hospital of Wenzhou Medical University, Wenzhou, 325000 P.R. China; 3 Shanghai Key Laboratory of Female Reproductive Endocrine-Related Diseases, Fudan University, Shanghai, 200011 P.R. China

**Keywords:** lncRNA, AOC4P, EMT, Metastasis, Ovarian cancer

## Abstract

**Objective:**

Currently, the function and mechanisms of long non-coding RNAs (lncRNAs) involved in the metastasis of epithelial ovarian cancer (EOC), especially those of the lncRNAs participated in the epithelial-mesenchymal transition (EMT) process, remains largely unknown. Here, we focused on a lncRNA named AOC4P and analysed its role in EOC.

**Materials and methods:**

The expression of AOC4P gene was examined with quantitative real-time quantitative PCR (qRT-PCR). The cell migration and invasion were detected by Transwell and scratch assays. The in vivo metastatic activity was evaluated by intraperitoneal metastasis model. The downstream genes were investigated by a tumour EMT real-time polymerase chain reaction (RT-PCR) array, and validated by qRT-PCR and Western blot.

**Results:**

The results showed that AOC4P expression levels were decreased in EOC tissues and cell lines, and that the under-expression of AOC4P was positively correlated with FIGO stage and lymph node metastasis. Furthermore, the knockdown of AOC4P expression in poorly metastatic EOC cell lines remarkably facilitated cell migration/invasion while the overexpression of AOC4P in highly metastatic EOC cell lines reduced the metastatic ability of these cells in vitro. Consistently, the anti-metastatic role of AOC4P in vivo was also verified by bioluminescence imaging and tumour dissection. Mechanistically, the anti-metastatic effect of AOC4P in EOC was partially mediated by the EMT process accompanied by the alterations in MMP9 and COL1A2 expression.

**Conclusion:**

These data highlight that AOC4P plays a critical role in EOC invasion/metastasis and could function as a novel and effective target for the lncRNA-based anti-metastatic clinical management of EOC.

## Introduction

Epithelial ovarian cancer (EOC), which represents the majority of all ovarian cancers (OC), has the highest mortality rate among gynecological cancers [1]. Poor patient prognosis is primarily attributed to the widespread metastatic events [2]. Due to easy invasion of adjacent organs or metastasis to the peritoneal cavity, the 5-year survival rate of OC patients remains the unsatisfactory rate of 30% [3]. Therefore, methods for elucidating the mechanisms underlying metastasis are urgently needed.

Epithelial-mesenchymal transition (EMT) exerts a major function in initiating metastasis [4]. Evidence indicates that at the initial stage of EOC metastasis, cells undergo EMT to acquire highly invasive phenotypes, and this acquisition is accompanied by multiple molecular events involving signalling pathways, epithelial and mesenchymal markers (EMT markers), EMT-transcription factors (EMT-TFs), and EMT-associated inducers such as matrix metalloproteinases (MMPs) [2, 5–8].

Apart from these well-known regulators, long non-coding RNAs (lncRNAs), a primary group of non-coding RNAs, are emerging as pivotal players in EMT-associated metastasis in malignancies [9]. The copper containing 4, pseudogene (AOC4P) is a recently discovered EMT-related lncRNA that was initially reported in hepatocellular carcinoma (HCC) [10]. Evidence has also shown the involvement of AOC4P in colorectal cancer (CRC) and gastric cancer (GC) [11, 12]. However, studies regarding the clinical value and biological role of AOC4P in EOC have not been elucidated.

In this study, we measured the expression of AOC4P in EOC clinical samples and cell lines, and analyzed the correlations between AOC4P expression and EOC clinicopathological features. Subsequently, to probe the metastatic function of AOC4P in vitro, three pairs of poorly/highly metastatic cell lines were chosen for loss/gain of function assays. Additionally, a nude mouse model of abdominal metastasis was established to determine the in vivo activity of AOC4P. Moreover, the molecular mechanisms underlying the AOC4P-mediated anti-metastatic effect on EOC were investigated, and experiments ascertained whether certain EMT-related factors were partially implicated in this phenotypic transformation associated with EMT. These data indicate the instructive value of AOC4P in EOC progress.

## Materials and methods

### Patients and samples

The study cohort consisted of 70 EOC patients and 10 patients with uterine fibroids who underwent hysterectomy and oophorectomy at the Obstetrics and Gynecology Hospital of Fudan University between August 2013 and September 2016. These 80 tissue samples (consisting of 70 EOC and 10 normal ovarian surface epithelial tissues) were collected by the Tissue Bank of the Obstetrics and Gynecology Hospital of Fudan University. And all samples were frozen immediately in liquid nitrogen and stored at − 80 °C until analysis. None of these patients had received preoperative treatment. This study was approved by the Ethics Committee of the Obstetrics and Gynecology Hospital of Fudan University (No. [2017]82).

### Cell lines and cell cultures

Three pairs of highly/poorly metastatic EOC cell lines (HEY-A8 and HEY, HO8910-PM and HO8910, and SKOV3-IP and SKOV3) were obtained from the University of Texas MD Anderson Cancer Center (Houston, TX, USA). The HEY-A8, HO8910-PM and SKOV3-IP cell lines are highly invasive EOC cell lines derived from the HEY, HO8910 and SKOV3 cell lines, respectively [13–19]. All cells were cultured in RPMI-1640 medium (Genom, Hangzhou, China) with 10% foetal bovine serum (FBS; Gibco, Grand, Island, NY, USA), 100 U/ml penicillin and 100 μg/ml streptomycin at 37 °C in a humidified atmosphere with 5% carbon dioxide.

### Establishment of stable AOC4P-knockdown and AOC4P-overexpression tumor cells

The lentiviral vectors, which were used to knockdown or overexpress AOC4P, were constructed by Genechem (Shanghai, China). The recombinant AOC4P-siRNA-1, full length of AOC4P and the negative control lentivirus were prepared, all of which were labeled with luciferase (luc-lentivirus). After 72 h, luciferin (0.15 mg/ml) was added into culture medium. To confirm luciferase expression of infected cells, they were then imaged under Bioluminescence Imaging Facility. Subsequently, positive cell clones were selected by puromycin for approximately 14 days to obtain the following new stable cell lines: HEY-luc-NC, HEY-luc-KD, HEY-A8-luc-NC, HEY-A8-luc-OE, HO8910-PM-luc-NC, H08910-PM-luc-OE, SKOV3-IP-luc-NC and SKOV3-IP-luc-OE.

### RNA extraction and quantitative real-time polymerase chain reaction (qRT-PCR)

Total RNA was extracted with TRIZOL reagent (TAKARA, Code No.9109, Dalian, China) and the isolated RNA was reverse transcribed to cDNA utilizing the Prime-Script™ RT Master Mix (TAKARA, RR036A, Dalian, China). The synthesized cDNA was subjected to qRT-PCR using SYBR Green qPCR (TAKARA, RR820A, Dalian, China). All reactions were performed according to the manufacturer’s instructions. The expression of target genes relative to that of the control GAPDH was determined by the calculation 2^−ΔΔCt^ method. The primer sequences are listed in Table 1.

**Table 1:** Primers used in the study

Gene	Primer	Sequence (5′-3′)
GAPDH	Forward	GTCATCAATGGAAATCCCATCA
	Reverse	CCAG TGGACTCCACGACGTAC
AOC4P	Forward	ACCAAGAGCCTGAAGACGA
	Reverse	TGCCTGTCACCAGAACACTA
NONHSAT076754	Forward	AAGTTTCTCACTCACCCACCTG
	Reverse	GAAGCATGTACAGTTCAGCATGTG
lncTCF7	Forward	AGGAGTCCTTGGACCTGAGC
	Reverse	AGTGGCTGGCATATAACCAACA
HEIRCC	Forward	GCTGCTATTCTGGTGCCC
	Reverse	TCAACTCCGATAAACAGGTGA
ZEB2NAT	Forward	GAG AGA CGA GAG ACC CTG AA
	Reverse	TGC ACA CAC CCT AAT ACA CAT
NKILA	Forward	AACCAAACCTACCCACAACG
	Reverse	ACCACTAAGTCAATCCCAGGTG
MMP2	Forward	GGAAGTCTGTGTTGTCCAGAGG
	Reverse	TGATTTGAAGCCAAGCGGTCT
MMP9	Forward	TCTACACCCAGGACGGCAAT
	Reverse	GAAGCCGAAGAGCTTGTCCC
COL1A2	Forward	CTTGAAAGCCTCAAAAGTGT
	Reverse	TCTCAGACCCAAGGACTATG
E-cadherin	Forward	GACGCGGACGATGATGTGAAC
	Reverse	TTGTACGTGGTGGGATTGAAGA
ZEB1	Forward	CCCTCTGGGATGCGAAACG
	Reverse	CTGCTTCTAGACAGGAAATCCCAC

### Cell transfection

A negative control small interfering RNA (siRNA-NC) and AOC4P-siRNAs (GenePharma, Shanghai, China) were used in combination with Lipofectamine 3000 transfection reagent (Invitrogen, Carlsbad, CA, USA) to transfect HEY, HO8910, and SKOV3 cells based on the manufacturer’s protocol. The target sequences of the AOC4P-siRNAs were 5′-GGUAAGGCAUCCUGGCAGAUU-3′ (siRNA-1), 5′-GGUCCUCUCUAUCUGUUUATT-3′ (siRNA-2). The sequence of siRNA-NC was 5′-UUCUCCGAACGUGUCACGUTT-3′. Knockdown efficiency was assessed by qRT-PCR after 48 h of transfection.

### Scratch assay

Seed the target cells on six-well plates. When the cells reached about 80–90% confluence, wounds were formed by draw a sterile 200 μL pipette tip. The scraped cells were washed with phosphate-buffer-solution (PBS), and serum-free media were added. The scratch area was imaged at a certain time interval (0 h, 24 h and 48 h) and cell migration capacity was calculated using fomula as follow: cell mobility = ((1- corresponding point scratch width)/initial scratch width) × 100.

### Cell migration assay

Cell migration abilities were assessed using Transwell chambers with a pore size of 8 μm (Millipore, Billerica, MA). The cells were loaded into the upper chamber with 200 μL of serum-free RPMI-1640 medium. The bottom Transwell chambers were filled with 600 μL of complete RPMI-1640 medium containing 10% FBS. 24 h later, the cells on the lower surface were fixed, stained and then five random fields (100×) were counted under a microscope.

### Cell invasion assay

The invasion assays were identical to the migration assay except that the Transwell membranes were pre-coated with Matrigel (BD Biosciences, Franklin Lakes, NJ, USA) as a matrix barrier before the cell incubation.

### In vivo intraperitoneal metastasis model

The protocols were approved by the Institutional Animal Care and Use Committee of Fudan University. Female 5- to 6-week-old Balb/c nude mice were obtained from Slac Laboratory Animal Co. Ltd. (Shanghai, China), maintained under a specific-pathogen free (SPF) condition and randomly distributed into two groups, the control group and the experimental knockdown group (six mice per group). The mice in the control group were intraperitoneally (i.p.) injected with HEY-luc-NC cells, while the mice in the knockdown group were injected with HEY-luc-KD cells (2× 106/0.2 ml). Then, the mice were monitored weekly at the Small Animal Bioluminescence Imaging Facility. Before imaging, each mouse was i.p. injected with luciferin (150 mg/kg) to stimulate luminescence. After 4 weeks, all mice were sacrificed, and the tumours were dissected for analysis.

### Tumour EMT RT-PCR array

After HEY cells were transfected with AOC4P-siRNA-1 or AOC4P-NC for 24 h, total RNA was extracted and then reverse transcribed into cDNA using an RT^2^ First Strand kit (Qiagen, Mississauga, ON, Canada). Based on the manufacturer’s protocol, a cDNA library was established and we analyzed the reverse transcribed cDNA with a Human Tumour Metastasis RT2 Profiler™ PCR array (Qiagen, Mississauga, ON, Canada), which contained 84 well known genes involved in the tumour EMT process. The expression fold-changes were calculated with the 2^-ΔΔCt^ method, and the results are exhibited.

### Western blot (WB) analysis

WB analysis was conducted as described previously [20]. The primary antibodies used were rabbit anti-GAPDH (Cell Signaling Technology, #5174, USA), anti-MMP9 (Abcam, ab76003, Cambridge, UK) and anti-COL1A2 (Abcam, ab, Cambridge, UK) antibodies.

### Data analysis

The data analysis was conducted using SPSS version 20.0 software (SPSS, Inc., Chicago, IL, USA) and GraphPad Prism software (Prism 5.0, GraphPad Software, San Diego, CA, USA). Continuous data were analyzed by Student’s t-test (2 groups) or one-way ANOVA (> 2 groups), while categorical data were analyzed by the χ2 test or Fisher’s exact test. *P* < 0.05 was defined as statistically significant.

## Results

### Downregulation of AOC4P expression is linked to advanced and aggressive OC clinical characteristics

**Fig. 1:**
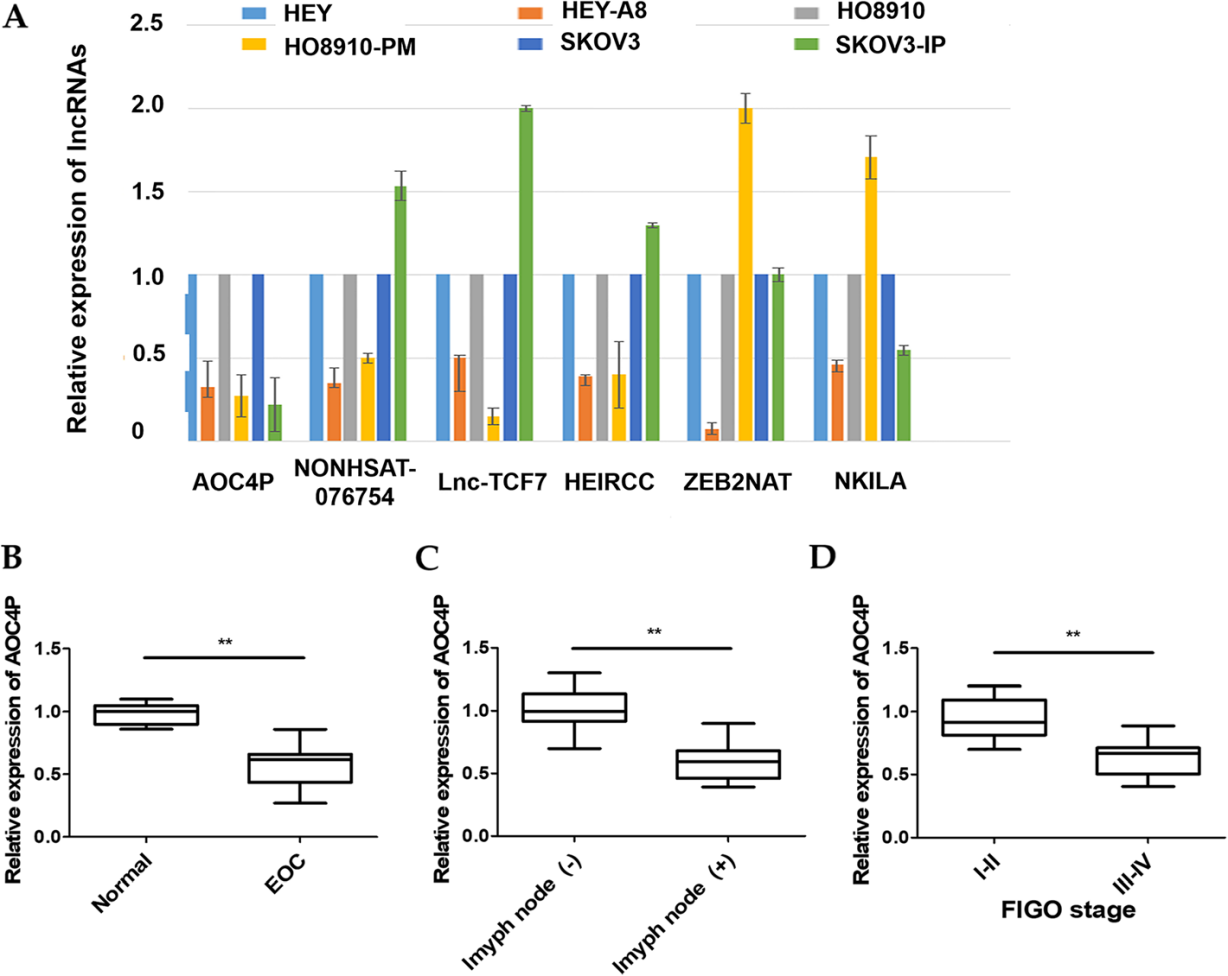
Identification of AOC4P among six EMT-associated lncRNAs and detection of AOC4P expression in EOC. **a** Expression analysis of six EMT-associated lncRNAs in highly metastatic EOC cell lines (HEY-A8, HO8910-PM, and SKOV3-IP) and their parental sublines (HEY, HO8910, and SKOV3). **b** Expression of AOC4P in EOC and normal ovarian tissues samples. **c** Expression of AOC4P in the lymph node (+) group and the lymph node (−) group. **d** Expression of AOC4P in the advanced FIGO stage (III-IV) cancer samples compared with that in the early FIGO stage (I-II) samples. Data are shown as the mean ± SD. **p < 0.01 vs. the NC groups

Based on existing studies [10, 11, 21–28], we chose six EMT-associated lncRNAs and investigated whether they are involved in EOC. Using highly metastatic EOC cell lines (HEY-A8, HO8910-PM, and SKOV3-IP) and their parental poorly metastatic sublines (HEY, HO8910, and SKOV3) as a cell model, we observed that AOC4P displayed an optimal consistency in expression levels and exhibited significantly lower levels in the highly metastatic cell lines than in the parental cell lines (Fig. 1a). Therefore, AOC4P were the focus of further clinical and functional explorations.

To further investigate the clinical implications of AOC4P in EOC, we tested AOC4P expression in 70 EOC and 10 normal ovarian surface epithelial tissues samples by qRT-PCR. The results showed that the AOC4P expression in the EOC tissue samples was significantly lower than that in the normal tissues samples (Fig. 1b). The expression levels of AOC4P in the lymph node metastasis-positive group (lymph node (+)) were significantly lower than those in the lymph node metastasis-negative group (lymph node (−)) (Fig. 1c). Additionally, relatively lower AOC4P expression levels were detected in the advanced FIGO stage (III-IV) cancer samples than in the early FIGO stage (I-II) samples (Fig. 1d). Moreover, to analyze the clinicopathological significance of AOC4P expression in EOC, the 70 EOC patients were divided into two groups, i.e., a high AOC4P expression group (*n* = 35) and a low AOC4P expression group (*n* = 35) based on the median value of relative AOC4P expression. Consequently, low AOC4P expression was positively associated with FIGO stage and lymph node metastasis, but no significant difference in other clinicopathological parameters such as age, histological subtype, residual tumour diameter, CA125 expression and ascites, were found (Table 2). These results illustrated that AOC4P has the potential to function as a tumour suppressor and might have an essential impact on EOC metastasis and aggressiveness.

**Table 2:** Correlation of AOC4P expression with clinicopathological characteristics in 70 EOC patients

Variable	Low AOC4P expression (*n* = 35)	High AOC4P expression (*n* = 35)	*P*-value
	n(%)	n(%)	
**Age (years)**			
< 50	14 (40.0)	10 (28.6)	
≥ 50	21 (60.0)	25 (71.4)	0.314
**Histological subtype**			
Serous	26 (80.0)	23 (62.9)	
Others	9 (20.0)	12 (37.1)	0.434
**FIGO Stage**			
I-II	2 (5.7)	8 (22.9)	
III-IV	33 (94.3)	27 (77.1)	0.040
**Residual tumour diameter (cm)**			
< 1	32 (91.4)	28 (80.0)	
≥ 1	3 (8.6)	7 (20.0)	0.172
**Lymph node metastasis**			
Absent	7 (20.0)	27 (77.1)	
Present	28 (80.0)	8 (22.9)	< 0.001
**CA125 level (U/ml)**			
< 600	10 (28.6)	12 (34.3)	
≥ 600	25 (71.4)	23 (65.7)	0.607
**Ascites**			
< 100	19 (54.3)	24 (68.6)	
≥ 100	16 (45.7)	11 (31.4)	0.220

### AOC4P interference facilitates EOC cell metastasis in vitro

**Fig. 2:**
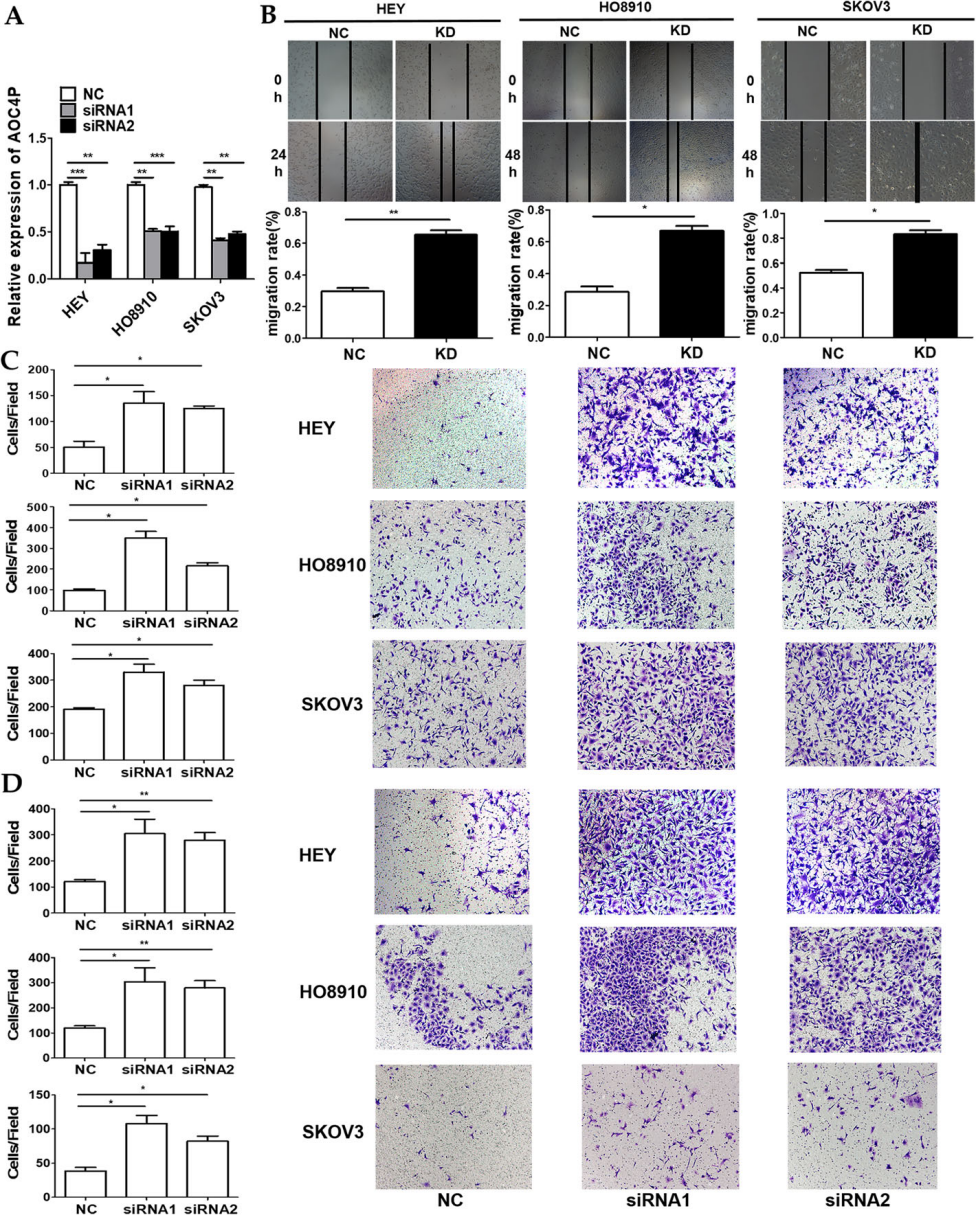
Knockdown of AOC4P promotes migration and invasion in HEY, HO8910 and SKOV3 cells. **a** The interference efficiency of AOC4P expression was determined by qRT-PCR. **b** Cell migration activities after AOC4P interference were assessed by wound healing assays. **c** Transwell migration assays were conducted to confirm the cell migration activity. **d** Cell invasion activities after AOC4P interference were assessed by transwell assays. Data are shown as the mean ± SD. * *p* < 0.05, ** *p* < 0.01, and *** *p* < 0.001 vs. the NC groups

Given that higher AOC4P expression was observed in the poorly metastatic EOC cell lines (HEY, HO8910, and SKOV3) (Fig. 1a), as well as the fact that lower expression levels of AOC4P were associated with aggressive tumour phenotypes in EOC, we speculated that AOC4P might exert a crucial function in EOC cell migratory and invasive abilities. To verify this hypothesis, a series of functional experiments in selected cells were conducted based on the siRNA-mediated knockdown of AOC4P expression. Initially, the interference efficiency of AOC4P expression was determined by qRT-PCR (Fig. 2a). Subsequently, tumour cell metastatic potential was evaluated in AOC4P-siRNA-transfected cells with Transwell and scratch assays. The results revealed that AOC4P interference remarkably facilitates the metastasis of HEY, HO8910, and SKOV3 cells in vitro (Fig. 2b-d).

### Overexpression of AOC4P inhibits EOC cell metastasis in vitro

**Fig. 3:**
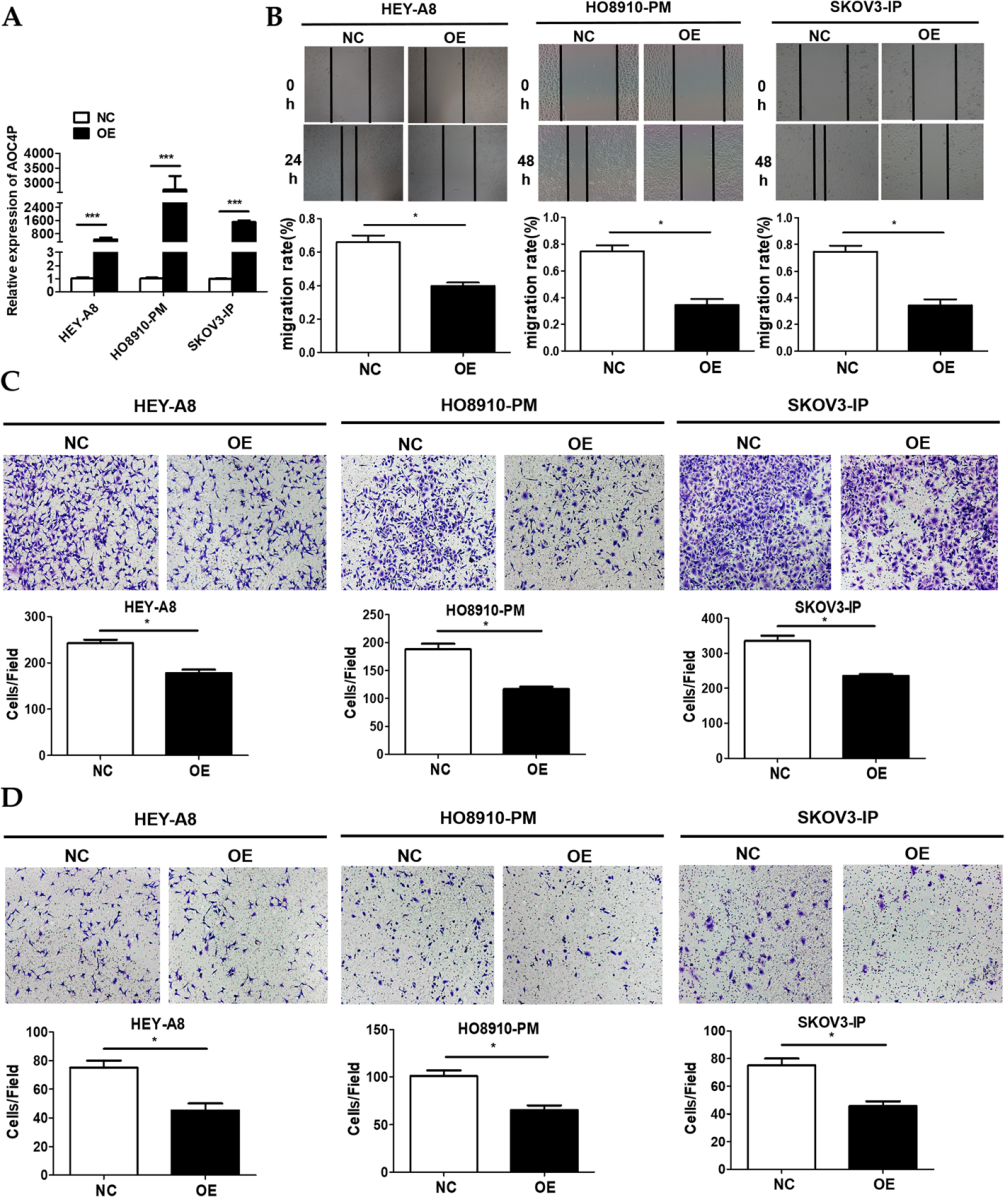
Overexpression of AOC4P hinders EOC cell metastasis in HEY-A8, HO8910-PM and SKOV3-IP cells. **a** Efficiency of AOC4P overexpression was confirmed by qRT-PCR. **b** Migration of AOC4P -overexpression cells was evaluated by wound healing assays. **c** Transwell migration assays were conducted to confirm the cell migration activity. **d** Invasion of AOC4P -overexpression cells was detected by transwell invasion assays. Data are shown as the mean ± SD. * *p* < 0.05, and *** *p* < 0.001 vs. NC groups

Since the loss-of-function assays determined that the knockdown of AOC4P expression facilitated EOC cell metastasis in vitro, we further investigated whether the overexpression of AOC4P would have the opposite effect on highly metastatic EOC cell lines (HEY-A8, HO8910-PM, and SKOV3-IP). We constructed stable AOC4P overexpression EOC cell lines (Fig. 3a) and, as expected, observed that overexpression of AOC4P significantly attenuated the metastasis of HEY-A8, HO8910-PM and SKOV3-IP cells in vitro (Fig. 3b-d). Collectively, these results indicated that AOC4P could inhibit EOC cell metastasis in vitro.

### AOC4P represses EOC metastasis in vivo

**Fig. 4:**
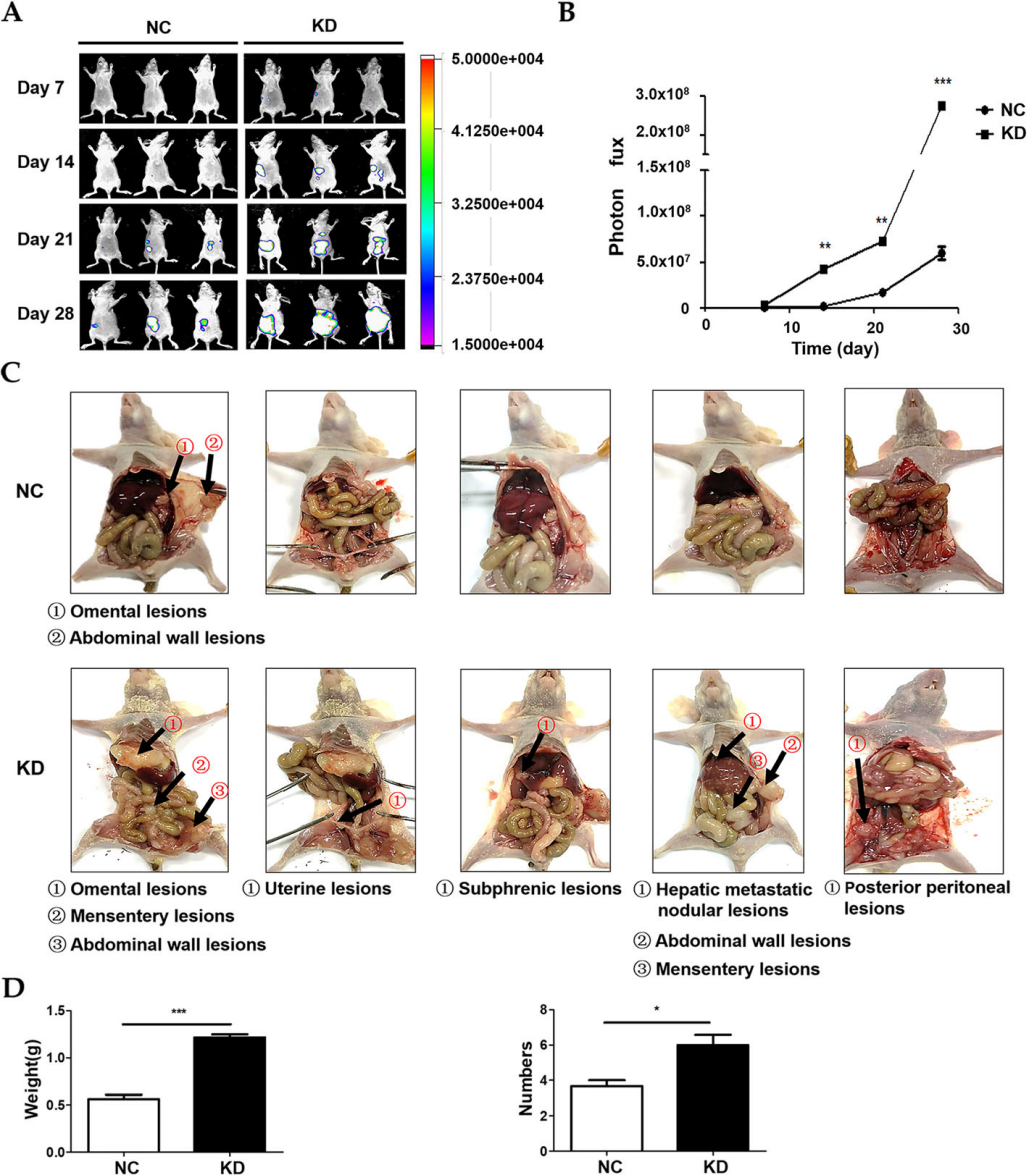
AOC4P suppresses metastasis in vivo. **a** Representative images of the luciferase signals in the HEY-luc-KD and HEY-luc-NC groups. **b** Quantification of the photon flux from the lesions detected by bioluminescence imaging. **c** Representative views of visible metastases in the peritoneal cavity. **d** Analysis of the numbers and weights of dissected tumours. Data are shown as the mean ± SD. * *p* < 0.05, ** *p* < 0.01, and *** *p* < 0.001 vs. the NC groups

To investigate the metastatic role of AOC4P in vivo, a nude mouse model of abdominal metastasis was established. The results gained from bioluminescence imaging showed that compared to that of HEY-luc-NC group, the luciferase signals of the HEY-luc-KD group were increased (Fig. 4a). The significant difference in photon flux quantification between the two groups was found early at 14 days after the abdominal injection and became more obvious at 21 and 28 days (Fig. 4b). Data analysis of the tumours dissected after 4 weeks revealed that the HEY-luc-KD group developed multiple visible metastatic nodules in the abdominal cavity, including abdominal wall lesions, omental lesions, mensenteric lesions, uterine lesions, subphrenic lesions, hepatic metastatic nodular lesions and posterior peritoneal lesions. In comparison, the HEY-luc-NC group exhibited limited tumour formation at the primary injection site in the abdominal wall or in the omental area near the injection site (Fig. 4c). Statistically, the HEY-luc-KD group showed more and larger metastatic nodules than the HEY-luc-NC group (Fig. 4d). Altogether, these results indicated that AOC4P could suppress EOC metastasis in vivo.

### AOC4P affects EOC metastasis via EMT-mediated gene alterations in MMP9 and COL1A2

**Fig. 5:**
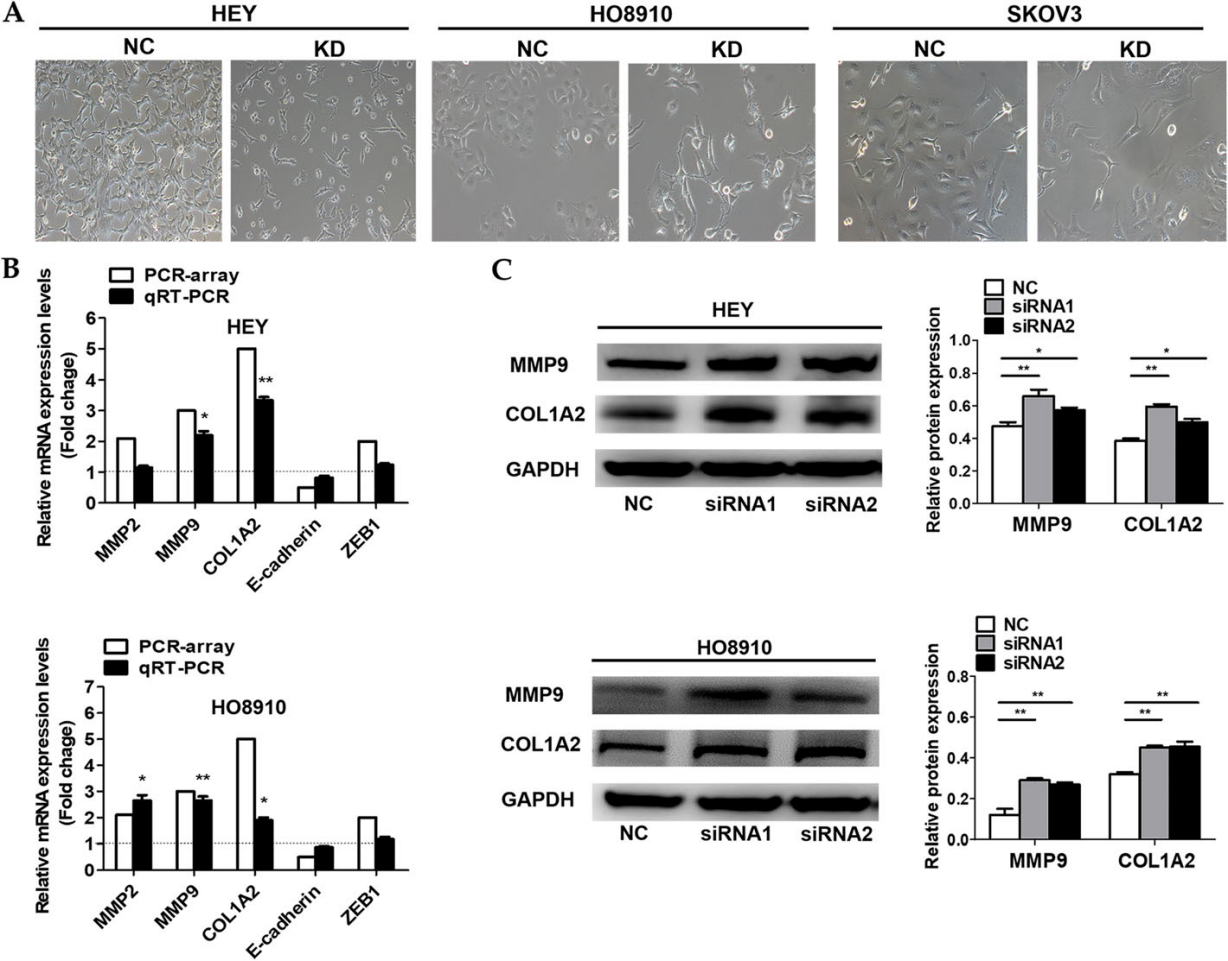
AOC4P suppresses EOC metastasis via the EMT-regulated downstream genes MMP9 and COL1A2. **a** Representative images of the morphology of the cells in AOC4P-siRNA or siRNA-NC groups are shown. **b** The expression levels of MMP9 and COL1A2 were detected by qRT-PCR in SKOV3 and HO8910 cells transfected with AOC4P-siRNA or siRNA-NC. **c** WB verification of MMP9 and COL1A2 expression in SKOV3 and HO8910 cells transfected with AOC4P-siRNA or siRNA-NC. Data are shown as the mean ± SD. * *p* < 0.05, and ** *p* < 0.01 vs. the NC groups

Since both the in vitro and in vivo data supported the anti-metastatic role of AOC4P in EOC, we explored the potential mechanisms involved. Interestingly, through observation of AOC4P-siRNA-transfected cells, we found that the knockdown of AOC4P resulted in the cells exhibiting a spindle-shape morphology and (or) starting to separate from one another, while the cells in the NC groups retained their morphology and cell–cell contact (Fig. 5a). Based on these phenomena, we hypothesized that EMT might participate in AOC4P-induced anti-metastatic activity in EOC. Initially, we performed an EMT RT-PCR array to explore whether the expression levels of certain EMT-related genes were changed after AOC4P interference. As a result, five genes were remarkably dysregulated (≥2-fold), with four genes (MMP2, MMP9, COL1A2, and ZEB1) upregulated and one gene (E-cadherin) downregulated (Table 3). Then, qRT-PCR and WB assays confirmed that MMP9 and COL1A2 was upregulated at both the mRNA and protein levels in two AOC4P-siRNA-transfected cells lines (HEY and HO8910) (Fig. 5b-c). Collectively, these data revealed that the two EMT-related genes MMP9 and COL1A2 might be involved in the AOC4P-mediated anti-metastatic effect.

**Table 3:** Differential expression of genes (≥2-fold) between HEY-AOC4P-siRNA-1 cells and HEY-siRNA-NC cells as identified by an EMT array

Gene name	GeneBank ID	Description	Function	Fold change
MMP2	NM_005343	Matrix metallopeptidase 2	Mesenchymal marker	2.1
MMP9	NM_004994	Matrix metallopeptidase 9	Mesenchymal marker	3.3
COL1A2	NM_000089	collagen type I alpha 2 chain	Mesenchymal marker	5.1
E-cadherin	NM_004360	Cadherin 1, type 1, E-cadherin (epithelial)	Epithelial marker	−2.0
ZEB1	NM_030751	Zinc finger E-box binding homeobox 1	Mesenchymal marker	2.0

## Discussion

EOC is recognized as the most malignant subtype of OC with uncomfortably poor prognosis [29]. The high rate of metastasis is acknowledged as chief culprit contributing to the poor outcome of EOC. Evidence indicates that EMT is one of the major molecular mechanisms inducing cancer metastasis [30] and is also an essential event in initiating metastasis of malignant tumours [31].

Mounting studies have elucidated the role of lncRNAs in a wide variety of human malignancies. In EOC, an increasing numbers of lncRNAs have been demonstrated to be related to metastasis, and several of these lncRNAs are considered to be EMT-related lncRNAs (e.g., HOTAIR, UCA1, MALAT1, CCAT1, Linc-ROR, H19, and NEAT1) [9]. Despite these advances, research on EMT-related lncRNAs has been limited in EOC. Therefore, it is worth gaining insight into novel EMT-related molecules, which may provide novel perspective for EOC diagnosis and therapy.

Using the literature, we identified six EMT-related lncRNAs (AOC4P, NONHSAT076754, Lnc-TCF7, HEIRCC, ZEB2NAT and NKILA), whose roles in EOC had not been investigated. Among them, AOC4P gained the most attention because the expression of AOC4P was consistently significantly lower in our highly metastatic cell lines (HEY-A8, HO8910-PM, and SKOV3-IP) than in the parental poorly metastatic cell lines (HEY, HO8910, and SKOV3). AOC4P, a recently identified lncRNA, was initially reported to suppress invasion and metastasis in patients with HCC [10]. Subsequently, it was reported that decreased expression of AOC4P was closely correlated with metastatic event and poor prognosis in patients with CRC [11]. Evidence also supported that AOC4P regulated tumor cell proliferation and invasion in GC [12]. However, no study has illustrated the involvement of AOC4P in EOC. Here, we found that the de-regulated expression of AOC4P was linked to aggressive clinicopathological variables including advanced FIGO stage and lymph node metastasis, suggesting that AOC4P might act as a tumour suppressor and could be applied as an additional biomarker in distinguishing less malignant and metastasis-prone outcome in EOC.

Recently, several lncRNAs have been identified to have downregulated expression and act as suppressive factors during the progression of multiple types of cancers. For instance, the silencing of SPRY4-IT1 leads OC cells to develop a more aggressive phenotype [32]. Low NKILA expression has been implicated in tongue squamous cell carcinoma invasion and metastasis [28]. For AOC4P, previous studies have revealed that the overexpression of AOC4P prominently reduced HCC and CRC cell metastasis. However, in GC, AOC4P was upregulated and promoted the migration and invasion of GC cells [10–12]. There was previous evidence that the expression levels of lncRNAs varied in various carcinomas with different tumor cell functions. For example, lncRNA BANCR was expressed differentially in GC and colon cancer [33, 34]. Inspired by these studies combined with our findings that decreased expression of AOC4P was correlated with lymph node metastasis and that tissue samples from the lymph node metastasis-positive group expressed lower AOC4P levels than those from the corresponding lymph node metastasis-negative group, we attempted to ascertain whether AOC4P was involved in EOC metastasis. As anticipated, Transwell and scratch assays showed that the depletion of AOC4P could promote migration and invasion in poorly metastatic EOC cell lines. In contrast, the overexpression of AOC4P impaired the metastatic and invasive potential of highly metastatic EOC cell lines in vitro. In line with the results of the in vitro assays, an in vivo experiment indicated that silencing AOC4P led to a remarkable increase in the number, weight and distribution of the tumour. These results, similar to its functions in HCC, indicate that AOC4P acts as a critical suppressor in EOC metastasis.

To date, the mechanisms underlying lncRNA-involved metastasis have not been fully elucidated. It is well-acknowledged that EMT is a critical initiating factor of tumour metastasis and invasion. Growing evidence has noted that EMT-related lncRNAs play critical roles in the progression of various types of tumours via diverse mechanisms [35]. Previous in mechanistic investigations have indicated that certain EMT regulators participate in AOC4P-mediated anti-metastatic behaviours [10–12]. Nonetheless, the molecular events underlying AOC4P-mediated anti-metastatic behavior of EOC are not elucidated. In the current study, we found that AOC4P-knockdown cells experienced morphological and distribution transformations from a cobblestone-like shape and (or) tight adhesion to spindle-shape morphology and (or) scattered cell–cell adhesion and these results agree with EMT features. Then, a tumour EMT RT-PCR array indicated that certain differentially expressed genes were identified after the knockdown of AOC4P expression. The subsequent verification of the results from the array confirmed that the expression of mesenchymal-associated factors including MMP9 and COL1A2 was upregulated. The changes in expression were validated at both the mRNA and protein levels, which further demonstrated that the EMT process is involved in AOC4P-mediated metastasis inhibition. Notably, certain EMT markers, such as E-cadherin, N-cadherin, vimentin, did not display significant difference in our study. Especially, the expression of E-cadherin was downregulated in data from tumour EMT RT-PCR array while it showed no difference in qRT-PCR verification in both two tumor cells (HEY and HO8910). According to the literature, there is evidence that the expression of E-cadherin displays no difference in several subtypes of OC [36]. In addition, between normal HCC cells and AOC4P-overexpressing HCC cells, there is no difference in E-cadherin expression [10]. The fact that we set the cut-off value of fold change as 2 when we analyzed the data from EMT PCR array, resulted in some differentially expressed EMT-related genes with fold change lower than 2 were ignored. Besides, although some typical EMT markers showed no difference in mRNA level in this manuscript, the protein level of these genes might be altered by the steps after transcription including post-transcriptional, translation and post-translational modification. In future work, WB assays are essential to be performed on these typical EMT markers to determine if they are differently expressed in protein level. Admittedly, there are some limitations of present study. First, adequate paraffin-embedded clinical tissue samples, which were not available in present study due to the limitation of our Tissue Bank, are needed for in situ hybridization (ISH) assays to further evaluate the expression and localization of AOC4P and better reveal the relevance of AOC4P to EOC. Second, more comprehensive and detailed work needs to be done to better unveil the biological processes AOC4P participated in, downstream genes AOC4P induced and the direct or indirect relationship between AOC4P and downstream genes.

## Conclusion

In conclusion, this study collected comprehensive data from the clinic, function and mechanism, and offers the first evidence of the role of AOC4P in EMT-involved metastasis in EOC. We show that the under-expression of AOC4P underlies the onset of EMT and aggressive metastasis in EOC by upregulating the expression of MMP9 and COL1A2. We have also shown that low expression levels of AOC4P are positively related with an advanced tumour stage and lymph node metastasis in EOC, suggesting that AOC4P could serve as an effective target for anti-metastatic strategies in EOC.

## Data Availability

Not applicable.
